# Autophagy and mitophagy in the context of doxorubicin-induced cardiotoxicity

**DOI:** 10.18632/oncotarget.16944

**Published:** 2017-04-07

**Authors:** Navid Koleini, Elissavet Kardami

**Affiliations:** ^1^ Institute of Cardiovascular Sciences, St. Boniface Hospital Albrechtsen Research Centre, Winnipeg, Manitoba, Canada; ^2^ Department of Physiology and Pathophysiology, Winnipeg, Manitoba, Canada; ^3^ Department of Human Anatomy and Cell Sciences, University of Manitoba, Winnipeg, Manitoba, Canada

**Keywords:** oncocardiology, anthracyclines, heart failure, impaired autophagy and mitophagy, lysosomal dysfunction

## Abstract

Doxorubicin (Dox) is a cytotoxic drug widely incorporated in various chemotherapy protocols. Severe side effects such as cardiotoxicity, however, limit Dox application. Mechanisms by which Dox promotes cardiac damage and cardiomyocyte cell death have been investigated extensively, but a definitive picture has yet to emerge. Autophagy, regarded generally as a protective mechanism that maintains cell viability by recycling unwanted and damaged cellular constituents, is nevertheless subject to dysregulation having detrimental effects for the cell. Autophagic cell death has been described, and has been proposed to contribute to Dox-cardiotoxicity. Additionally, mitophagy, autophagic removal of damaged mitochondria, is affected by Dox in a manner contributing to toxicity. Here we will review Dox-induced cardiotoxicity and cell death in the broad context of the autophagy and mitophagy processes.

## OVERVIEW OF DOX-INDUCED CARDIOTOXICITY

Doxorubicin (Dox), a non-selective class I anthracycline antibiotic, is a potent chemotherapeutic agent which is used for the treatment of numerous cancers [1]. The use of Dox is limited, however, due to serious side effects; the most prominent is cardiotoxicity which can manifest acutely as well as years after treatment has been discontinued leading to left ventricular dysfunction, dilated cardiomyopathy and heart failure [2–4]. Toxic effects of Dox include cardiomyocyte damage and apoptotic and necrotic cell death [5]. The severity of heart disease is linked to accumulated Dox dosage during the course of the anticancer therapy ranging from 3-5% in patients that received a cumulative dose of 400mg/m^2^ to 18-48% in patients receiving 700mg/m^2^ (and 100% of mice receiving 71mg/m^2^) [1, 6, 7].

Dox is a mitochondrial toxin, and mitochondrial damage is central to Dox-induced cardiac dysfunction and cell death [8]. Cardiomyocytes require large numbers of healthy-functioning mitochondria to ensure sufficient ATP production for contractile function and cell survival. Dox-induced over-production of reactive oxygen and nitrogen species (ROS and RNS) such as superoxide, hydrogen peroxide, hydroxyl radical and peroxynitrite has been proposed to be the main cause of Dox-induced acute cardiomyocyte toxicity and cell death by damaging various molecular constituents and organelles [9–12]. A detailed description of the pathways by which Dox induces production of ROS and RNS can be found in references [13, 14]. Nitric oxide synthase (NOS) and nicotinamide adenine dinucleotide phosphate-oxidase (NOX) enzymes play an important role in Dox-induced production of ROS [15]. Dox can be reduced to a semiquinone by NOX and/or NOS leading to the production of superoxide and hydrogen peroxide, and subsequently the highly reactive hydroxyl radical [14]. NOS enzymes catalyze the production of nitric oxide from N-arginine; superoxide can react with nitric oxide producing peroxynitrite which is highly reactive oxidizing DNA, proteins, and peroxidizing lipids. Both endothelial NOS (eNOS) and inducible NOS (iNOS) isoforms have been implicated in mediating Dox toxicity via the production of RNS [16]. Deletion or overproduction of eNOS in mice was shown, respectively, to improve or worsen cardiac outcome post-Dox compared to wild type groups [17]. There is also some evidence that iNOS, which is increased by Dox administration, contributes to the production of RNS; iNOS-knockout mice showed protection from Dox-induced toxicity [18]. Dox also interacts with iron, and Dox-iron complexes contribute to further hydroxyl radical production. Hydroxyl radical damages DNA and proteins and extensively peroxidizes lipids which contribute to major cellular damage and death [19].

Dox has a robust attraction to negatively charged membranes such as the inner mitochondrial membrane causing lipid peroxidation [10, 20]. Dox-induced peroxidation of cardiolipin uncouples respiratory chain complexes at mitochondria, resulting in reduced ATP and increased ROS production [21–24], and promoting formation of the of mitochondrial permeability transition pore, mPTP. Formation of mPTP causes cytochrome C release and induction of an intrinsic pathway of apoptosis; it is also an important feature of necrotic cell death [25]. Despite multiple studies and strong evidence from many experimental models that anti-oxidant therapies can decrease or prevent Dox-induced cardiotoxicity, results have not been as promising in the clinical setting [26–28]. Additional mechanisms of Dox-toxicity need to be considered and targeted.

Dox kills rapidly proliferating cancer cells by intercalating with their DNA and forming covalent adducts which lead to the inhibition of DNA polymerase and nucleic acid synthesis. Additionally, Dox cross-links Topoisomerase IIα (TOPIIα) to the DNA, forming a TOPIIα-Dox-DNA complex resulting in DNA breakage and cell death, a major mechanism for killing cancer cells which express high levels of TOPIIα [29–33]. Unlike tumor cells, cardiomyocytes do not express TOPIIα, rather they express topoisomerase IIβ (TOPIIβ) which can also form DNA-TOPIIβ-Dox complexes, cause double-strand breaks and cell death [34, 35]. Dox-induced DNA damage, mediated by TOPIIβ significantly alters the nuclear and mitochondrial transcriptome and markedly decreases mitochondrial biogenesis [36, 37]. There is strong evidence that TOPIIβ is a major contributor to Dox-induced cardiotoxicity; transgenic mice lacking TOPIIβ were not susceptible to Dox cardiotoxicity in an acute as well as chronic setting [37]. Interestingly, the compound dexrazoxane which is in clinical use for preventing Dox-induced cardiomyopathy due to its iron-chelating and anti-oxidant properties[38], belongs to the molecular category of TOPII poisons. It has been suggested that the TOPIIβ inhibitory activity, rather than the iron-chelating action, of dexrazoxane confers protection of cardiomyocytes from Dox [39].

Dox induced DNA damage leads to the activation of the Ataxia-Telengiectasia Mutated protein which binds to DNA break sites and upregulates and activates the tumor suppressor protein p53 [40]. Upregulation of p53 by Dox has been linked to increased ROS and double strand DNA damage, and there is considerable support to the notion that p53 protein is mediating Dox-induced apoptotic cell death of cardiomyocytes in vitro and in vivo [41–43]. Anthracycline-induced p53 up-regulation is reported to suppress transcription of GATA-4, a cardioprotective transcription factor [44]. Nevertheless, p53-independent pathways to Dox-induced deleterious effects in cardiomyocytes, such as apoptotic cell death, oxidative and nitrosative stress, and cardiac fibrosis have also been documented, in a mouse model of cardiomyocyte-specific p53 knockout [45]. In addition, using a juvenile model of Dox toxicity, Zhu and colleagues reported antithetical roles for p53 in the acute versus chronic response to Dox: the expression of dominant-interfering p53 in the heart resulted in short-term (1 week) protection against Dox, but worse outcome, compared to wild type mice at 13 weeks post -Dox [46]. Overall, it would appear that the role of p53 in mediating the multiple Dox-induced deleterious effects is context dependent, and likely depends on the model used and the timing of the assessment.

**Figure 1:**
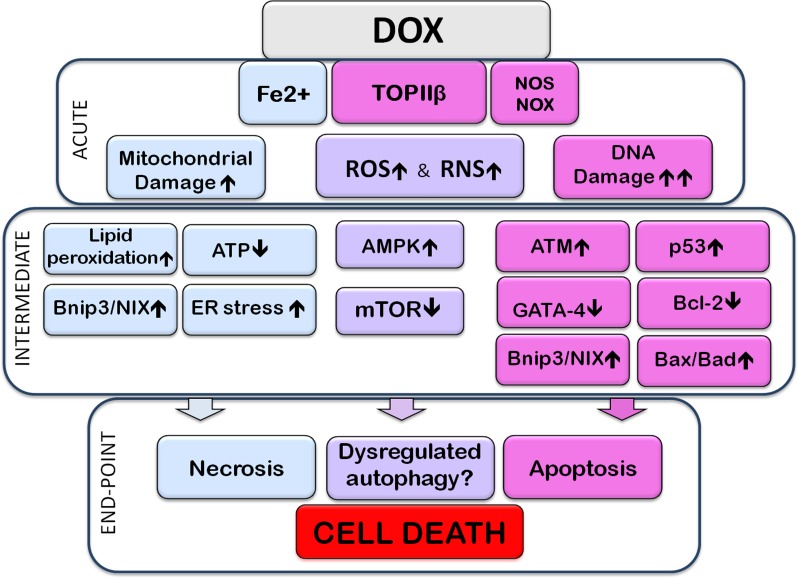
Subcellular events associated with Doxorubicin-induced cardiotoxicity The image highlights acute, intermediate, and end-point (cell death) events resulting from exposure to Doxorubicin (Dox). The acute section illustrates the direct interaction of Dox with subcellular entities; the intermediate section illustrates direct consequences of these interactions. Events within the acute, or intermediate sections are likely to occur simultaneously and cross-talk with each other. The end-point section is meant to show that the preceding events lead to apoptotic, necrotic, and/or dysregulated autophagy-associated cell death. Acute Events: Dox, upon entering the cell, interacts directly with molecules and organelles: interaction with topoisomerase-IIβ (TOPIIβ) leads to DNA damage. Interaction with nitric oxide synthase (NOS), nicotinamide adenine dinucleotide phosphate-oxidase (NOX) and Fe2+, promotes reactive oxygen or nitrogen species stress (ROS or RNS, respectively), contributing to further DNA damage, oxidation and nitrosylation of proteins and peroxidation of lipids. Dox binds to mitochondrial DNA and impairs the electron transport chain resulting in production of ROS and decreased ATP. Fe2+-Dox complexes are toxic to mitochondria and the endoplasmic reticulum (ER), by causing, for example, lipid (including cardiolipin) peroxidation. The DNA damage response activates the ataxia telangiectasia mutated (ATM) protein which upregulates and activates the tumor suppressor p53. P53 upregulates expression of pro-apoptotic members of the Bcl-2 family such as Bax and Bad; it also increases expression of Bcl2/adenovirus E1B 19 kDa protein-interacting protein 3 (Bnip3), which can cause mitochondrial damage and necrotic cell death, as well as initiate mitophagy. DNA damage and increased levels of ROS lead to downregulation of the transcription factor GATA-4 which decreases expression of the anti-apoptotic, and anti-autophagy-initiation protein, Bcl-2. P53 can also inhibit the activity of mammalian target of Rapamycin (mTOR) signaling, thus dis-inhibiting autophagy initiation. Some studies have indicated that Dox can elicit AMP-activated kinase (AMPK) activation. Activation of AMPK, resulting from reduced ATP levels, can inactivate mTOR and initiate autophagy. Dox-induced effects on ROS and RNS production, mitochondrial and ER damage, DNA and gene expression, culminate in the promotion of apoptotic and necrotic cell death. Dysregulation of the autophagy/mitophagy processes are also linked to Dox-induced cell death. Signals/events associated mostly, although not exclusively, with apoptotic or necrotic cell death are included, respectively, in pink or pale-blue boxes. Pale-purple boxes contain signals/pathways associated with various types of subcellular dysfunction, including autophagic dysregulation.

An important effector of Dox-induced cell death is Bnip3 (BH3-only protein Bcl-2-like 19 kDa-interacting protein-3), a member of the Bcl-2 family of proteins. There is evidence that Dox-induced cardiotoxicity is mediated by upregulation of Bnip3 and induction of necrosis in cardiomyocytes [47]. A similar protein, Bnip3L/NIX, an effector of apoptosis, was also found to be upregulated in the heart and myocytes by Dox, in vivo and in vitro, in response to Dox-induced downregulation of miR-30 [48]. To show the crucial role of Bnip3, transgenic mice were created expressing a mutant form of Bnip3 which is unable to insert to mitochondrial membranes; these mice were resistant to Dox induced cardiotoxicity [47]. Bnip3 causes depolarization of mitochondria by promoting mPTP formation which leads to cell death [47]. Both p53 and Bnip3 are also considered to regulate autophagy [47], as will be discussed in the following section. Dox treatment promotes premature ageing of cardiomyocytes, in vitro (long-term cultures) and in vivo [49, 50]. It is of interest that ageing is characterized by decreased potential for autophagy and mitophagy, and this in turn contributes to the pathology of ageing [51]. One might therefore surmise that Dox-induced chronic changes would, as in ageing, also include reduced ability for autophagy. This was shown to be the case by Hoshino and colleagues [52], who reported decreased autophagic elimination of damaged mitochondria (mitophagy) in models of ageing as well as during Dox-induced cardiotoxicity in vitro and in vivo. Figure 1 shows a broad overview of the mechanisms implicated in Dox-cardiotoxicity

## AUTOPHAGY

Autophagy is a conserved process aimed at maintaining cell and tissue homeostasis under normal as well as stress conditions, including nutrient starvation, changes in metabolism, and energy and oxygen status. Autophagy can be subdivided to micro-autophagy, chaperone mediated autophagy, and macro-autophagy; the latter refers to the sequestration of cytosolic cargo by double-membrane bound vesicles, with subsequent fusion with lysosomes for degradation [53]. The term autophagy as used here refers to macro-autophagy. The overall Dox-induced cellular changes such as the accumulation of oxidized and damaged macromolecules and organelles as well as significantly reduced mitochondrial capacity for ATP production would be expected to trigger autophagy.

**Figure 2:**
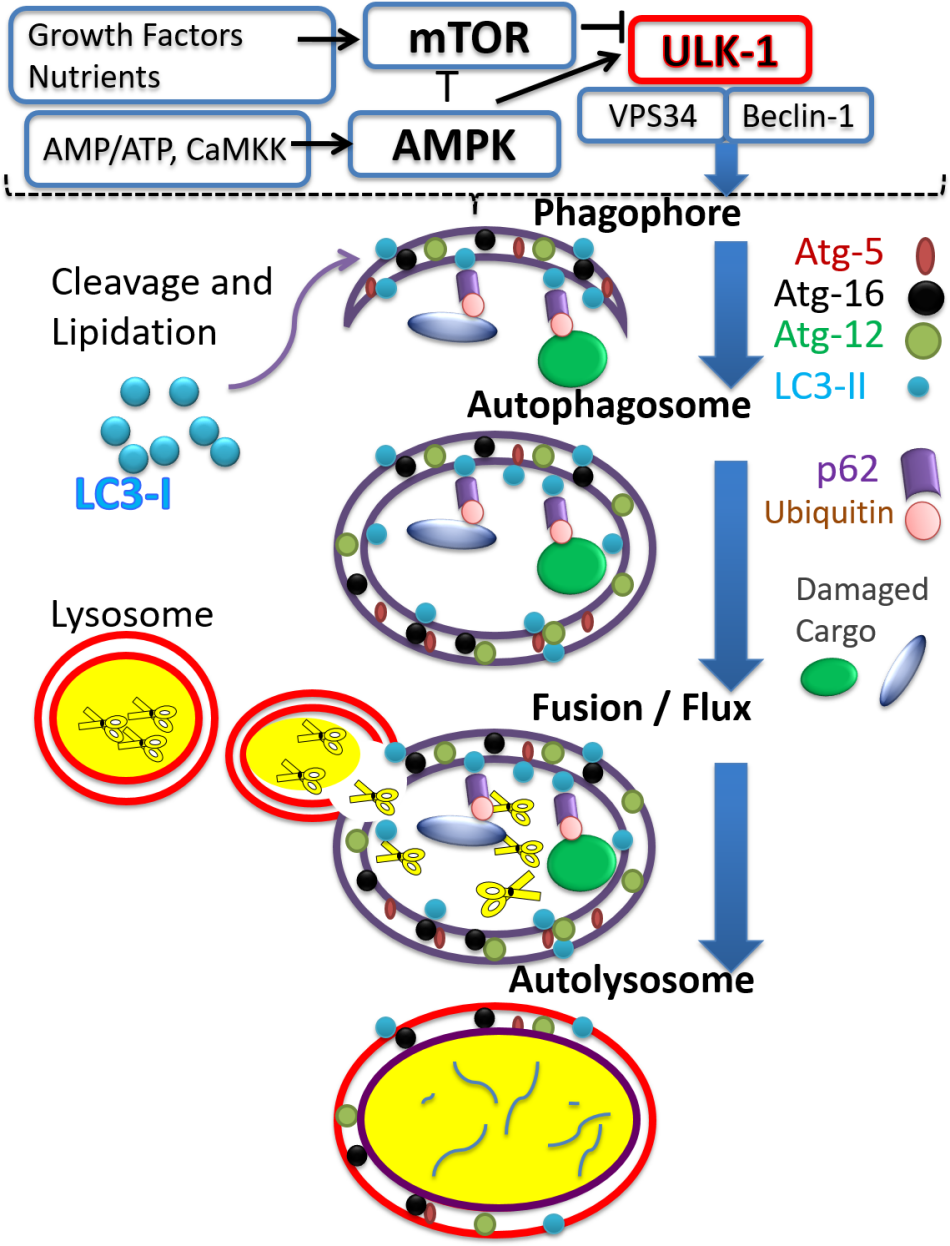
The autophagy process The figure illustrates major steps constituting the process of autophagy, aiming at recycling targeted cargo. Vertical blue arrows point to a progression from autophagy-initiation via the activated unc-51-like autophagy activating kinase 1 (ULK-1) complex, to the formation/elongation of membranous entities such as the phagophore; to the formation of the autophagosome around the cargo, followed by fusion with the lysosomes and formation of autolysosomes where cargo is degraded. Major signaling pathways regulating the initiation of autophagy are included in the upper portion, separated from the rest of the figure by the broken line, and include the mammalian target of rapamycin (mTOR) and AMP activated kinase (AMPK) pathways. Activators of mTOR, include growth factors and high nutrient status, while activation of AMPK occurs when AMP/ATP ratio increases, and also upon increased calcium activating calcium/calmodulin-dependent protein kinase kinase 2 (CaMKK). The figure shows the antithetical action of mTOR versus AMPK on autophagy initiation, as manifested by ULK-1 activation. ULK-1 mediated activation of Beclin-1 and vesicle-mediated vacuolar protein sorting 34 (VPS34) complex initiates a cascade of events leading to phagophore formation. A few representative proteins associated with the autophagy process are included for each step. Autophagy related gene (Atg) proteins 5, 12, and 16 incorporation elongates the formed phagophore. LC3-II is formed by proteolytic cleavage and lipidation of microtubule-associated proteins light chain 3B (LC3-II) which anchors it to the phagophore. Ubiquitination tags damaged cargo which can then interact with the ubiquitin binding protein, p62/SQSTM1, and, via interaction of the latter with LC3-II, cargo is engulfed within the autophagosome. For completion of the process, fusion of autophagosomes with lysosomes is required, to form autolysosomes. In the autolysosomes cargo and associated proteins are digested by the various degradative lysosomal enzymes, symbolized by scissor images.

The autophagy process is composed of several steps. Initiation of autophagy describes the formation of the isolation membrane and phagophore, which then expands to engulf the cargo (protein aggregates or damaged organelles), thus forming the autophagosome. The process is completed by autophagosome clearance which occurs after fusion with lysosomes enabling degradation of cargo by lysosomal enzymes as illustrated in Figure 2. Degradation by-products, such as amino acids, can then be re-used for the building of new macromolecules or for meeting metabolic demands [54].

Several autophagy related genes (Atg) and signal transduction pathways have been implicated in autophagy initiation and progression, and have been described in some detail in recent reviews [55]. Autophagic initiation requires the phosphorylation of the protein Beclin-1 by ULK-1 (activated unc-51-like autophagy activating kinase 1), with subsequent activation of VPS34 (vesicle-mediated vacuolar protein sorting 34) complexes. VPS34 is a class II PI3 kinase, increasing phosphatidyl inositol phosphate (PI3P), involved in the recruitment of other Atg proteins, phagophore formation and elongation [56–58]. The LC3 (light chain 3 microtubule associated protein) is converted to LC-3I after proteolytic cleavage of its C-terminus; LC3-I is then lipidated, becoming LC3-II, which associates with the double membrane of the autophagosome. LC3-II acts as a ‘hook’ for proteins possessing an LC3-II-interacting domain (LIR), recruiting them to the autophagosome. One of these proteins is the scaffold protein p62/SQSTM1, which possesses both a LIR domain and ubiquitin-binding domain [59]. p62/SQSTM1 attracts ubiquitinated aggregates to the autophagosome, and therefore facilitates their eventual degradation. Completion of the autophagy process, autophagosome clearance following fusion with the lysosome, results in the degradation of p62/SQSTM1 by lysosomal enzymes. Increased accumulation of p62/SQSTM1, on the other hand, is linked to the inhibition of autophagic flux/clearance [59]. Other proteins can act in a manner similar to p62/ SQSTM1. These include the “neighbor of BRCA1 gene 1” (NBR1) [60]; the TNF-receptor-associated factor 2 (TRAF2) [61], and the Smad ubiquitin regulatory factor 1, SMURF1 [62].

Lysosomal associated membrane proteins (LAMPs) are essential for recognition and fusion with autophagosomes; mutations in LAMP2 (Danon's disease) are associated with incomplete autophagy and accumulation of autophagosomes, resulting in systemic problems, including hypertrophic cardiomyopathy in patients [63].

The most upstream signaling component of the autophagy process is represented by the ULK-1 kinase complex [64]. This complex is under tight control of two major signaling pathways, mammalian target of Rapamycin (mTOR) and AMP activated protein kinase (AMPK). While mTOR inhibits, AMPK activates ULK-1 [58]. Both of these pathways are affected by Dox, although there are some conflicting data, especially regarding the role of AMPK activation.

AMPK is a central sensor of cell energetic status [65]. Low cellular energy levels, increased ROS, and elevated intracellular Ca^2+^ lead to the phosphorylation and activation of AMPK [66]. Additionally, some cytokines (such as adiponectin) [67, 68] and growth factors (such as vascular endothelial growth factor, VEGF) have been shown to activate AMPK, by activating an upstream phosphorylating enzyme, LKB-1 (Liver Kinase B-1) [68, 69]. Activated AMPK is capable of inducing autophagy by de-repressing mTOR-mediated autophagy inhibition [70].

The serine/threonine kinase mTOR, activated downstream of growth factors and receptor tyrosine kinase signaling, is a highly conserved kinase considered to act as a master regulator of cell growth, metabolism, and protein synthesis, and to inhibit autophagy initiation. The mTOR forms two different signaling complexes (mTORC1 and mTORC2) [71]. The mTORC1 is a point of convergence for autophagy regulatory signals: it binds to the ULK-1 complex (phosphorylating ULK-1 at serine 757) and prevents the ULK-1-mediated phosphorylation of Beclin-1. As mentioned earlier, Beclin-1 phosphorylation is required for autophagy initiation; thus mTORC1 negatively regulates autophagy initiation [57, 58]. Activated AMPK can phosphorylate ULK-1 complex at Ser317 and Ser 777. These phosphorylations are required for autophagy initiation [70]. A schematic representation of the process of autophagy is shown in Figure 2.

## DOX AND THE AUTOPHAGY PROCESS

### Stimulation of autophagy prior to Dox is protective

A number of studies have indicated that boosting autophagy prior to the administration of Dox can protect from Dox toxicity. Administration of Rapamycin, an mTOR inhibitor which is used to stimulate autophagy, rescued mice from Dox-induced cardiomyopathy [72]. In aging rats, moderate caloric restriction in combination with resveratrol administration prior to Dox (6 weeks) stimulated autophagy, and protected against subsequent Dox toxicity [73]. Moderate diet restriction in rats, a process expected to stimulate autophagy, protected from acute Dox-injury [74]; in mice, starvation for 2 days upregulated autophagy and protected against Dox [75]. It is anticipated that modest activation of autophagy would eliminate dysfunctional mitochondria which are responsible for increased ROS production, remove any toxic aggregates and generally boost cellular health before Dox insult.

### Dox-induced effects on the autophagy process are deleterious

While the boosting of autophagy before Dox appears to be cardioprotective, post-Dox autophagy-associated signaling likely contributes to Dox-induced toxicity and presents a complex, not fully understood picture. Nevertheless, strategies aimed at preventing post-Dox autophagy-initiation and autophagosome formation have been shown to be protective. Pharmacologic inhibition of post-Dox autophagy by 3-methyladenine (a class III PI3-kinase inhibitor) or the silencing of Beclin-1 or Atg-5 protected cardiac cells from Dox toxicity [11, 76–80].

There is emerging consensus that Dox induces cellular changes consistent with autophagy initiation and autophagosome formation in cardiac cells, shown by a variety of approaches. Transmission electron microscopy evaluation in H9C2 cells indicated that Dox increases prevalence of autophagic vacuoles [11]. Dox-induced accumulation of autophagic vacuoles has been shown in mouse [75] and rat hearts [81]. Increased LC3-II levels and upregulation of autophagy related genes have been reported [11, 68, 76–80, 82–85]. Immunofluorescence-based detection of ‘dots’ (autophagosomes) containing either endogenous LC3-II, or overexpressed GFP-LC3 also pointed to the upregulation of autophagosome formation or accumulation, in *in vitro* and *in vivo* studies [11, 68, 77, 79, 82, 85]. In some models, however, decreased relative levels of lipidated LC3-II in response to Dox were reported [86–88]. Dox induced up-regulation of Beclin-1 and Atg-5 has been corroborated by several studies [68, 77, 80]. Atg-5 upregulation in mouse hearts and primary cardiomyocytes furthermore is attributed to the overproduction of 4-Hydroxynonenal, 4-HNE through lipid peroxidation, [76].

The effect of Dox on specific signals associated with various steps of the autophagy process has been addressed in several studies. AMPK and mTOR signals are considered to play antithetical roles, with AMPK triggering and mTOR inhibiting autophagy initiation [66, 71]. Conflicting reports exist on the effect of Dox on AMPK activation, summarized in Table 1. Several groups, using in vitro and in vivo systems, have reported AMPK activation post-Dox [11, 58, 89, 90]. Decreased cardiac AMPK activity post-Dox has also been described [75, 91–95], while no changes in AMPK activation post-Dox have also been reported [96, 97]. It is possible that the effect of Dox on AMPK is transient or cyclical, and dependent on a multiplicity of parameters including dosage and duration of treatment with Dox and experimental models used. The effects of age, sex and even circadian rhythm may also need to be considered [98].

**Table 1:** The effects of Doxorubicin on AMPK (AMP-activated kinase)

*In vitro*				
Cell Type	Dox Dosage	Time post-Dox	AMPK	Reference
Neonatal rat cardiomyocytes	1 μM	2 hours	**↑**	[89]
Neonatal rat cardiomyocytes	1 μM	6 - 24 hours	**↓**	[91]
H9C2	0.17 - 1.71 μM	2 hours	**↑**	[90]
H9C2	10 μM	10-30 minutes	**↑**	[11]
H9C2	0.25 - 2 μM	16 hours	**↓**	[92]
H9C2	2 μM	6-24 hours	**↓**	[93]
H9C2	10 nM	72 hours	No change	[97]
***In Vivo***				
**Species**	**Dox Dosage**	**Weeks post-Dox**	**AMPK activity**	**References**
Rat (male, 8-10 weeks)	20 mg/kg Intraperitoneal (IP), single dose	2	**↑**	[89]
Rat (8-10 weeks)	15 mg/kg	1	↓	[93]
Rat (male, 260-280g)	15mg/kg (3 doses) IP, 1 week interval	2	↓	[91]
Rat (male, adult, 300-400g)	18 mg/kg, 6 doses, over two weeks, IP	After the last injection	↓	[94]
Rat (male, 300g)	6 injections over 2 weeks for a total dose of 12 mg/kg, IP	4	↓	[95]
Mouse (male)	15 mg/kg, IP	12 days	No change	[58]
Mouse (adult)	Total of 20 mg/kg in 2 doses, IP	5 days after the first injection	↓	[75]
Mouse (male, 6-8 weeks, 20-22g)	15 mg/kg, for 6 days, IP	4	No change	[96]
**Species**	**Dox Dosage**	**Weeks post-Dox**	**AMPK mRNA**	**Reference**
Rat (male, 4 weeks, 200 g)	2.5 mg/kg every 48 hours, repeated six times, IP	3 weeks after the first injection	No change	[128]
	3 mg/kg once a week for four weeks, Intravenous	6 weeks after first injection	↓	
Mouse	IP: 15 mg/kg		**↑**	[129]

There is strong evidence based on in vitro and in vivo studies in rat, mouse and rabbit models that Dox inhibits mTOR, an inhibition that is expected to contribute to cardiomyocyte injury [11, 90, 99–102], possibly by causing an exacerbated autophagy-initiation response, summarized in Table 2. Dox-induced mTOR inhibition has been linked to p53 upregulation. Mice expressing cardiomyocyte-restricted dominant-interfering p53 are resistant to Dox induced cardiotoxicity, and retain normal levels of active mTOR, at least in the acute setting. Additionally, mice expressing cardiomyocyte-restricted constitutively active mTOR were found to be resistant to Dox insult [101]. Another group reported that p53 upregulation led to increased Bnip3 in cardiomyocytes [43]; knock-down of Bnip3 or dominant-negative inhibition of Bnip3 abrogated p53-induced autophagy.

**Table 2:** The effect of Doxorubicin on mTOR (mammalian target of rapamycin)

*In vitro*				
Cell Type	Dox Dosage	Hours post-Dox	mTOR activity	Reference
Neonatal rat cardiomyocytes	1 μM	48	**↓**	[130]
H9C2	0.2 μM	12	**↓**	[99]
H9C2	0.52 μM	12-24	**↓**	[11]
*In Vivo*				
**Species**	**Dox Dosage**	**Days post-Dox**	**mTOR activity**	**Reference**
Mouse (male, 6-8 weeks)	4 mg/kg	16	No change	[131]
Mouse (adult)	20 mg/kg	7	**↓**	[101]
Mouse	12 mg/kg	28	**↓**	[102]

Another pathway by which Dox can promote autophagy initiation may be through the p53-mediated suppression of the transcription factor GATA-4 and the resulting down-regulation of the pro-survival protein Bcl-2. Bcl-2 binds to Beclin-1 and thus prevents it from interacting with VPS34, and from initiating autophagy [56, 79]. Dox can also promote Bcl-2 phosphorylation which inhibits the Bcl-2/Beclin1 interaction again facilitating autophagy initiation [42].

Overall Dox affects a number of signaling pathways converging to a robust initiation of autophagy and stimulation of autophagosome formation. For the autophagy process to elicit a protective effect, however, it is important that the process is completed through autophagosome clearance. In this context, cargo recognition/tagging (by ubiquitin) and lysosomal degradation are important steps. Thus it is important to examine how Dox affects ubiquitination, the p62/ SQSTM1protein, and the lysosomes.

It has been widely confirmed that Dox significantly increases the total levels of ubiquitinated proteins in cardiomyocytes [72, 75, 78, 82, 85, 86]. The ubiquitinated cargo can interact with p62/ SQSTM1 and facilitate lysosomal targeting. Whether Dox-induced hyper-ubiquitination is due to massive damage to intracellular proteins, dysregulation in signaling pathways of the ubiquitination process, or because of decreased proteasomal degradation is still to be elucidated [103]. It is interesting to note that Dox affects various types of ubiquitin ligases in heart cells. DOX upregulates the muscle specific ubiquitine ligase MuRF 1 (Muscle RING finger 1), and also muscle atrophy F-box (MAFbx)/atrogin-1 which contributes to its cardiotoxic properties [104], while decreasing the expression of Parkin, another E3 ubiquitin ligase which is important for mitophagy [84].

Several groups have examined the effect of Dox on the accumulation of p62/ SQSTM1, with conflicting results. Increased p62/ SQSTM1 levels in the hearts of rat and mouse models post-Dox (1-6 days) have been reported [73, 75, 105]; a decrease in p62/ SQSTM1 has been observed post-Dox [77, 84]. A biphasic response has also been described, consisting of increases in p62/ SQSTM1 at earlier time points post-Dox, followed by decreased or baseline levels at later time points [85, 106]. These differences may reflect differences in Dox dosage, sex, different strains of animals, different cell types for in vitro studies, and most importantly, different treatment duration and end-points. This issue needs to be investigated further, because measuring p62/ SQSTM1 levels post-Dox is used to document effects on autophagy clearance/flux and completion of autophagy.

There is an emerging consensus, strengthened by recent studies, that Dox inhibits autophagic flux, by inhibiting lysosomal biogenesis and/or lysosomal function [72, 75, 88, 105, 106]. Bartlett and colleagues demonstrated that Dox decreases expression of TFEB (transcription factor EB) which regulates the expression of genes related to autophagy and lysosomal biogenesis [88]. Decreased TFEB expression in cardiomyocytes led to proteotoxicity and cell death; restoration of TFEB on the other hand restored flux and prevented cell death[88]. Using an experimental model of moderate Dox-induced toxicity, Li and colleagues showed that Dox-induced early upregulation of LC3-II was caused by inhibition of autophagic flux; furthermore, Dox was shown to promote autolysosome accumulation and prevention of lysosomal acidification in vivo [106]. Using tandem green fluorescence (GFP)-red fluorescence (RFP)-LC3 constructs in rat neonatal ventricular cardiomyocytes, the same groups showed that Dox inhibited autophagic flux in vitro. It is of note that while both reports implicated the lysosomes in causing inhibition of autophagic flux, one group did not see any changes in lysosome number per se [106], while the other reported decreased lysosome accumulation due to Dox [88]. This difference likely reflects methodological issues with the use of Lyso-Tracker dye which detects acidic organelles; the increase in lysosomal pH caused by Dox [106] may have led to an underestimation of lysosomes, and autolysosomes, by Lyso-Tracker Red [88, 106]. Regardless of the precise mechanism, impaired autophagic flux caused by Dox, would be expected to promote increased ROS and proteotoxicity.

By attenuating autophagy-initiation through the use of Beclin-1 haplo- insufficient mice Li and colleagues [106] were able to prevent Dox-induced toxicity, illustrating the detrimental role of autophagy initiation post-Dox. An important observation from the work by Li and colleagues was that Dox-induced inhibition of autophagic flux was observed only within a certain time period post-Dox, and not at later time points such as 4 weeks post-Dox [106]. Thus discrepancies between studies regarding the effects of Dox on autophagy may be cause by “lack of evaluation of autophagy as a process of flux”, using instead “snapshot-in time” approaches [106]. Tandem GFP-RFP-LC3 (“green” and “red”) fluorescence reporter approaches are increasingly used to monitor the movement of LC3-II from autophagosome to autolysosome, based on differences in fluorescence caused by differences in pH. The neutral environment of the autophagosome would allow both GFP and RFP fluorescence, while the acidic environment of autolysosome would only allow RFP, “red”, fluorescence. Because, however, Dox was shown to inhibit lysosomal functionality by preventing acidification, reliance on the tandem GFP-RFP-LC3 approach may lead to underestimation of lysosomal, and autolysosomal presence post-Dox [88, 106]. Thus complementary approaches are required to monitor the various steps of the autophagy process after Dox administration.

The use of inhibitors such as Bafilomycin A1 (prevents fusion of autophagosome to the lysosome) and Chloroquine (inhibits lysosomal activity), introduces additional interpretation issues. By blocking lysosomal degradation of cargo these reagents can indicate whether changes in the levels of ‘classic’ autophagy markers (LC3-II and p62/ SQSTM1 for example) are due to an effect on de novo autophagosome formation or on autophagosome cargo degradation [59]. However, the timing of administration, as well as dosage used (sufficient for inhibition but not toxic), need to be standardized, and off-target effects need to be taken into account for meaningful comparison between studies. A detailed description of problems associated the use of lysosomal inhibitors can be found in [104].

Liposomal formulations of Dox have been used to reduce Dox accumulation and toxicity on healthy tissues, and there is evidence from clinical applications that this approach may reduce heart injury [107, 108]. To our knowledge, there are no reports as to how liposomal Dox would affect autophagy in the heart. One may speculate that since heart muscle- associated Dox concentration is reduced when Dox is administered in liposomes [109], Dox-induced deleterious effects, including dysregulated autophagy, would be reduced.

### MITOPHAGY AND DOX

Myocardial contractile function and cell survival depend on a high content of healthy mitochondria to ensure sufficient ATP production through oxidative phosphorylation. Defective mitochondria are targeted for autophagic elimination (mitophagy), a process which contributes to the mitochondrial quality control system. Mitophagy plays a fundamental role in cardiomyocyte survival under various cellular stresses. Additional and possibly overlapping processes involved in mitochondrial quality control include the ubiquitin/proteasome-mediated elimination of mitochondrial proteins; and the elimination of toxic mitochondrial contents via formation of mitochondrial-derived vesicles (MDV) [110]. The latter is reported to occur more frequently than mitophagy under baseline conditions, and to represent a fast response to Dox-induced oxidative stress, preceding mitophagy [86].

Various mechanisms mediating the mitophagy process have been described, and reviewed recently in references [111–114]. Specific proteins at the mitochondrial outer membrane can act as selective mitophagy ‘receptors’, by possessing the LIR motif which binds LC3-II and enables mitochondrial engulfment by the autophagosome [115]. Two major pathways to mitophagy have been described for cardiomyocytes, the PINK1/Parkin pathway, and a Parkin-independent pathway, the Bnip3/Nix pathway. Several Parkin-independet mitophagy pathways have been described for various cell types but have not been identified in the cardiac system.

#### PINK1/Parkin

Parkin-mediated mitophagy occurs in damaged/depolarized mitochondria: depolarization of the outer mitochondrial membrane promotes the stabilization of PINK1, a mitochondrial serine/threonine kinase, at the mitochondrial outer membrane. PINK1 binds to Parkin, facilitating its translocation to mitochondria. Moreover, PINK1 phosphorylates ubiquitin, which is required for the ubiquitin ligase activity of Parkin [113]. Once recruited to mitochondria, Parkin ubiquitinates mitochondrial surface proteins such as Mitofusin 1, 2 (Mfn1, 2), voltage dependent anion channel-1 (VDAC1), and MIRO, a GTPase enzyme facilitating mitochondrial transport [116]. Parkin-dependent ubiquitination of Mfn and MIRO leads to proteasome degradation of these proteins and prevents mitochondrial re-fusion, keeping mitochondria in a fragmented state, stopping mitochondrial movement and promoting mitochondrial autophagic removal [113, 114, 117].

Expression levels of Parkin/PINK1, taken as indicators of mitophagy potential, were reported to show a biphasic response post-Dox in mouse hearts [118]: decreased Parkin/PINK1, at 5 days post Dox suggested decreased Parkin-mediated mitophagy at this time point. At 2 weeks post-Dox, however, expression of these proteins bounced back and reached levels even higher than those of untreated controls. Increased expression of a protein, Fis1, associated with mitochondrial fission and increased mitophagy, was also observed [118]. There is some evidence that the mitochondrial fission and mitophagy inhibitor peptide mdivi-1 prevented the deleterious effects of Dox[119], which would suggest that excessive mitophagy contributes to Dox-toxicity.

Another group has reported that the Dox-induced increase in p53, which can interact with and sequester Parkin to the cytosol, resulted in decreased Parkin translocation to mitochondria and decreased mitophagic potential, which was proposed to contribute to Dox-toxicity by allowing the accumulation of damaged mitochondria. This effect of Dox was not observed in p53-defficient mice, and could be counter-acted by over-expression of Parkin in neonatal rat cardiomyocytes [52]. It should be noted that Hoshino and colleagues [52] measured the mitophagy response after stimulation with carbonyl cyanide m-chlorophenylhydrazone (CCCP), which represent potential for, rather than endogenous, mitophagy.

#### Bnip3 and Bnip3L/NIX

Two members of BH3-only family proteins, Bnip3 and Bnip3L/NIX which have been associated with apoptotic and necrotic cell death, and the induction of permeability transition, can also act as mitophagy receptors. These proteins contain an LIR motif which enables them to interact with LC3-II on the surface of the autophagosomes. Unlike Parkin, the translocation of Bnip3 to mitochondria does not require loss of membrane potential. The ability of Bnip3 to induce permeability transition pore opening and cell death can be distinct from its ability to promote autophagy; these outcomes are likely modulated by distinct phosphorylation states. The phosphorylation of serines 23 and 17, adjacent to the LIR domain, was reported to promote mitophagy [120]; on the other hand, phosphorylation of C-terminal Bnip3 residues can block cell death, without affecting autophagic elimination [120]. Currently, it is not known how Dox affects Bnip3 or Bip3L/NIX- dependent mitophagy in cardiomyocytes. Following Dox, Bnip3 is reported to translocate to mitochondria and promote permeability transition and depolarization [47]. Depolarization would be expected to recruit Parkin to mitochondria. In fact, the Bnip3-mediated mitophagy is reported to depend on Parkin recruitment in cardiomyocytes [121].

**Figure 3:**
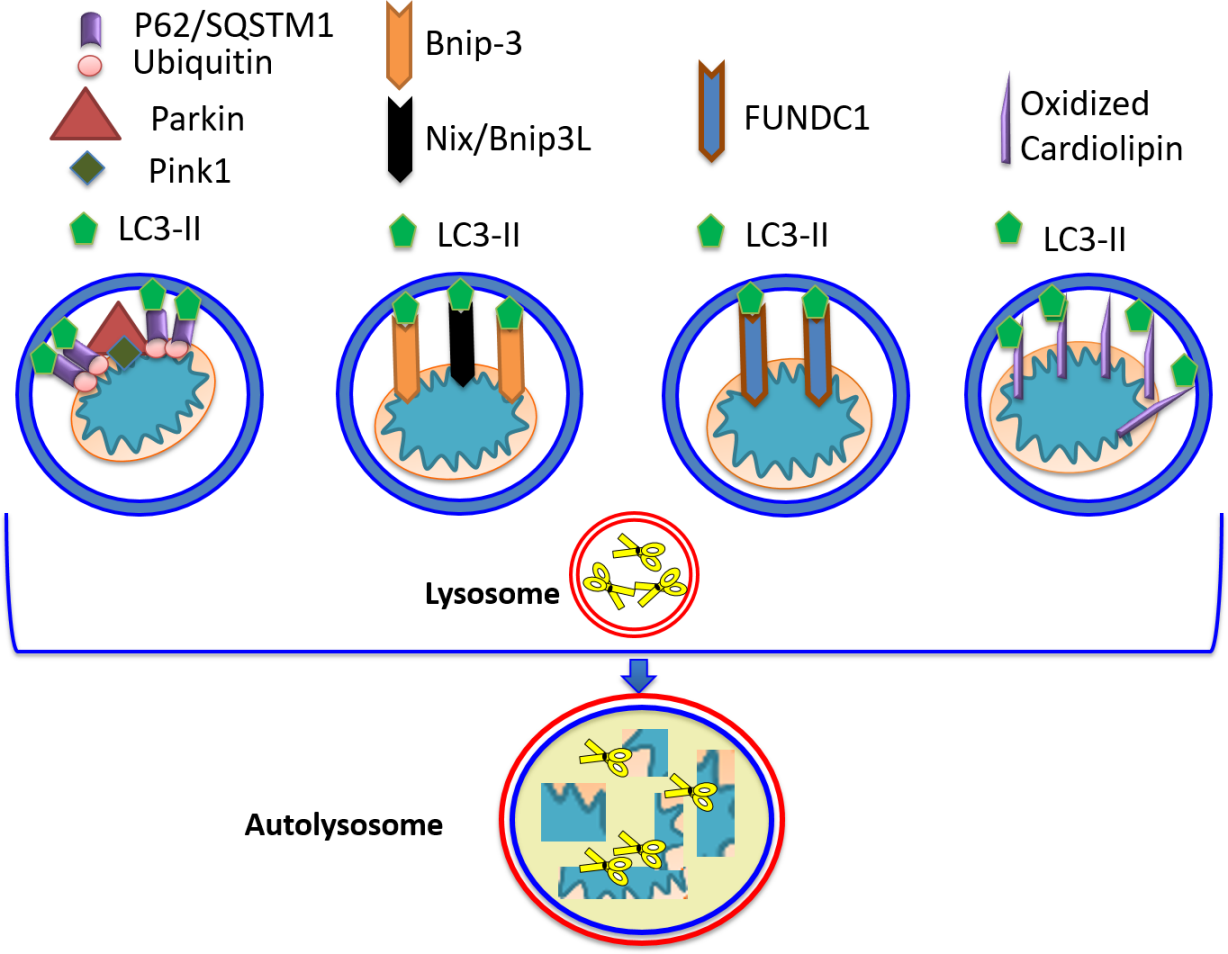
Mitophagy receptors Four pathways leading to targeting/ recognition of mitochondria for autophagic elimination have been described. The Parkin/PINK1 pathway, operating on depolarized mitochondria, consists of PINK1 stabilization, enabling interaction with Parkin and its translocation to the outer mitochondrial membrane, OMM. Parkin can ubiquitinate various OMM proteins enabling recognition and interaction with p62/SQSTM1. LC3-II can interact with p62/SQSTM1 allowing autophagosomal engulfment of mitochondria, and subsequent degradation via fusion with the lysosome. In the second pathway, Bcl2/adenovirus E1B 19 kDa protein-interacting protein 3 (Bnip3) can also act as mitophagy receptor, as it possesses the LC3-II recognition motif, as well as a transmembrane domain which anchors it to mitochondria. Nix/Bnip3L (the other member of Bnip3 family) acts in a similar fashion. Translocation of Bnip3 to mitochondria does not require loss of membrane potential. Additional molecules possessing LC3-II-interacting ability include FUN14 Domain Containing 1 (FUNDC1), and peroxidized cardiolipin.

FUNDC1, an outer mitochondrial membrane protein with an LIR motif enabling it to interact with LC3-II has also been linked to mitophagy, although only hypoxia-induced mitophagy has been studied; the role of Dox on FUNDC1 mediated mitophagy remains to be determined [113, 114]. Cardiolipin, which is induced by Dox to translocate to the outer mitochondrial membrane, could also serve as another mitophagy ‘receptor’. An illustration of the various mitophagy receptors is shown in Figure 3. A member of the Bcl-2 family of proteins, Bcl-2 like-13, possessing a LIR motif, was also identified in mammalian cells as a mitophagy receptor [122].

There is currently some controversy as to the relevance of the PINK1/Parkin pathway in cardiac baseline or stress-induced mitophagy [123, 124]. A notion that may explain differences between studies is the possibility that different mitophagy receptors could target different mitochondrial populations within the myocytes. Adult cardiomyocytes possess subsarcolemmal (SSM) and interfibrillar mitochondria (IFM), with distinct morphological and functional properties as well as distinct sensitivities to calcium overload [125]. There is currently limited information regarding how these different mitochondrial populations may be targeted for mitophagy, before or after Dox. Recently, a group studied the effects of Dox on SSM and IFM in adult female rats [126]. It was reported that SSM accumulated more Dox than IFM; furthermore, Dox resulted in an increase in PINK1 and a decrease in p62/SQSTM1 in the IFM population only, while Parkin content was not changed in either SSM or IFM. The authors interpreted the results as Dox-induced upregulation of Parkin mediated mitophagy in IFM [126]. However, one can speculate that Dox mediates inhibition of Parkin translocation to the IFM with impaired subsequent p62/SQSTM1 translocation as has also been described in [52].

The relative contribution of the various mitophagy receptors to baseline and Dox-associated mitophagy remains to be determined. One promising approach would be to use the Keima-mouse, expressing a mitochondria-targeted form of the fluorescent protein Keima [127], to examine how mitophagy is affected post-Dox. Fluorescence of the Keima protein is pH sensitive and changes color in the acidic environment of the lysosomes. This mouse model has already demonstrated that baseline levels of cardiac mitophagy are high relative to other organs [127], and could provide valuable information about Dox-associated mitophagy over time, within myocytes and in the various regions of the heart. The mt-Keima mouse could also address the effect of various pre-conditioning, cardioprotective manipulation on the mitophagy process before and after Dox-administration.

## CONCLUDING REMARKS

**Figure 4:**
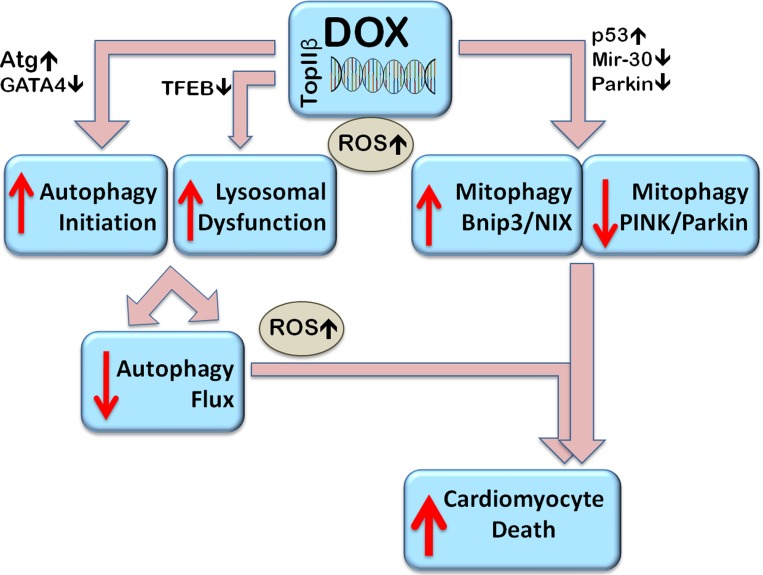
Proposed mechanisms of Dox-induced dysregulation of autophagy and mitophagy resulting in cell death Dox stimulates autophagy initiation (by upregulating Atg genes, for example) and at the same time, by decreasing expression of the master transcription factor EB (TFEB), impairs function of available lysosomes and prevents their biogenesis resulting in autophagosome accumulation and inhibition of flux. Autophagosome accumulation contributes to the accumulation of reactive oxygen species (ROS). Dox induced changes in gene expression include the downregulation of Parkin and via p53, in impairment of its translocation to mitochondria, thus inhibiting Parkin/PINK1 mediated mitophagy. Parkin-independent mitophagy, such as Bcl-2-like 19 KDa-interacting protein-3 (Bnip3) - mediated mitophagy can be upregulated. Overall, the dysregulated autophagy and impaired mitophagy can induce cardiomyocyte death.

There is no doubt that a systematic approach will be required to elucidate the role of autophagy and mitophagy in Dox-induced pathology, taking into account potential sex and ageing- related effects. Our overall conclusions based on available evidence are summarized in Figure 4. One can surmise that Dox leads to an over-activation of autophagy initiation while at the same time preventing autophagy completion due to deleterious effects on lysosomes. The resulting accumulation of un-degraded cargo which promotes ROS overproduction would be expected to be toxic to the cells, contributing to cell death. It would seem that Dox dysregulates endogenous mitophagy, and MDV production, in a manner that contributes to Dox toxicity. Since Dox promotes Parkin depletion in a manner similar to the ageing process one can conclude that Dox may selectively diminish Parkin/PINK1-mediated mitophagy. At the same time, Dox-induced Bnip3, and Bnip3L/NIX upregulation would suggest that Bnip3/NIX receptor mediated mitophagy takes over, contributing to Dox-cardiotoxicity.

## Abbreviations

Dox: Doxorubicin
ROS: reactive oxygen species
RNS: reactive nitrogen species
NOS: nitric oxide synthase
iNOS: inducible nitric oxide synthase
eNOS: endothelial nitric oxide synthase
NOX: nicotinamide adenine dinucleotide phosphate-oxidase
mPTP: mitochondrial permeability transition pore
TOPIIα: Topoisomerase-II α
TOPIIβ: Topoisomerase-II β
ATM: ataxia telangiectasia mutated
Bnip3: Bcl2/adenovirus E1B 19 kDa protein-interacting protein 3
Atg: autophagy related genes
ULK-1: unc-51-like autophagy activating kinase 1
VPS34: vesicle-mediated vacuolar protein sorting 34
PI3P: phosphatidyl inositol phosphate
LC3: light chain 3 microtubule associated protein
p62/SQSTM1: p62/Sequestosome-1
NBR1: neighbor of BRCA1 gene 1
TRAF2: TNF-receptor-associated factor 2
SMURF1: Smad ubiquitin regulatory factor 1
LAMP: Lysosomal associated membrane protein
mTOR: mammalian target of Rapamycin
AMPK: AMP activated protein kinase
VEGF: vascular endothelial growth factor
MuRF: Muscle RING finger
MAFbx: muscle atrophy F-box
TFEB: transcription factor EB
MDV: mitochondrial-derived vesicles
Mfn: Mitofusin
VDAC1: voltage dependent anion channel-1
MIRO: Mitochondrial Rho GTPase
CCCP: carbonyl cyanide m-chlorophenylhydrazone
SSM: subsarcolemmal mitochondria
IFM: interfibrillar mitochondria
mt-Keima: mitochondria keima.
